# Radiosensitization of Clioquinol Combined with Zinc in the Nasopharyngeal Cancer Stem-like Cells by Inhibiting Autophagy in Vitro and in* Vivo*

**DOI:** 10.7150/ijbs.40305

**Published:** 2020-01-14

**Authors:** Yuan Ke, Chaoyan Wu, Yifei Zeng, Mengge Chen, Yonghong Li, Conghua Xie, Yunfeng Zhou, Yahua Zhong, Haijun Yu

**Affiliations:** 1 Department of Radiation and Medical Oncology, Zhongnan Hospital of Wuhan University, Wuhan, China; 2 Hubei Key Laboratory of Tumor Biological Behaviors, Wuhan, China; Hubei Cancer Clinical Study Center, Zhongnan Hospital of Wuhan University, Wuhan, China; 3 Department of Integrated Traditional Chinese Medicine and Western medicine, Zhongnan Hospital of Wuhan University, Wuhan, China

## Abstract

Loco-regional recurrence of nasopharyngeal carcinoma (NPC) after radiation therapy is one of the main types of treatment failure. This study is aimed to explore the possible causes of inside-field recurrence of NPC patients in order to develop effective treatment methods. Our study indicated that CD44 and autophagy proteins in tumor tissues of patients with recurrent NPC are higher than that of the relapse free patients. The *in vitro* experiments further confirmed that cancer stem cells (CSCs) were more radioresistant with enhanced autophagy activity. Treatment with clioquinol (CQ) combined with zinc could obviously enhance the radiosensitivity of CNE-2s cells through autophagy inhibition, activation of the caspase system and impairment of DNA damage repair. The* in vivo* experiments have further consolidated our findings. Our results suggest that CSCs and enhanced autophagy activity may be involved in the inside-field recurrence of NPC, and CQ combined with zinc could be an important therapeutic approach for recurrent NPC.

## Introduction

Radiotherapy is the primary treatment for nasopharyngeal carcinoma (NPC) patients due to its anatomic location and radiosensitivity 1. Clinically, posttreatment recurrence and distance metastases are still obstacles to successful treatment of NPC cases. Therefore, management of inside-field recurrent NPC patients remains a huge clinical challenge.

Some studies speculate that the main reason for the relapse and metastases of NPC may be the presence of cancer stem-like cells (CSCs) 2-4. CSCs tend to repopulate malignant tumors during radiotherapy and accelerated tumor repopulation is a major cause of radiotherapy failure 5. In addition, there are also studies shown that CSCs contribute to radioresistance through preferential activation of autophagy and an increase in DNA-repair capacity 6, 7. Although such assumption and conclusion are based on strong clinical and experimental rationality, the identity of these stem cells and the nature of their interaction with ionizing radiation (IR) remain obscure.

Recently, autophagy has been shown to help in acquisition of resistance in CSCs towards anticancer therapy in various cancers 8, 9. There are studies shown that autophagy plays an important role in the stem maintenance 10, DNA damage repair 11, 12 and radioresistance^13^. Increased autophagy activity can cause resistance to IR 14. Therefore, targeting autophagy in CSCs may aid to enhance the radiosensitivity of nasopharyngeal cancer and reduce tumor recurrence or metastasis. However, the therapeutic promise is not to be verified experimentally yet.

Clioquinol (CQ), a Chloroquine analogue, autophagy inhibitor which prevent autophagosome maturation, has been shown to increase the anticancer activity of various chemotherapeutic drugs in different cancer cells 15-17. And we have previously found that CQ could target zinc to lysosome, leading to alterations of lysosome integrity and lysosome-mediated apoptotic cell death 18. Moreover, CQ can also suppress NF-κB activity, which is more pronounced in the presence of zinc 19. However, the role of CQ combined with zinc to IR in nasopharyngeal cancer stem cell is not yet reported.

The aim of this study is to explore the causes of inside-field recurrence of NPC patients after definitive radiotherapy. We observed that the presence of stem cells and increased autophagy activity played an important role in this process. Subsequently, we identified the possibility of radiosensitization of CQ combined with zinc in NPC stem-like cells, and explored the molecular mechanisms which were implicated in this process.

## Materials and Methods

### Patients selection

This study was approved by the Zhongnan Hospital of Wuhan University Ethics and Scientific Committee (2019040). All patients had signed written informed consent. The inclusive criteria were as follows: (1) histologically confirmed NPC before first treatment, (2) primarily treated with concurrent chemo-radiotherapy (CCRT) or IMRT alone with or without neoadjuvant/adjuvant chemotherapy, (3) finished the entire course of definitive radiotherapy, (4) received re-biopsy and confirmed by the pathological diagnosis for the local recurrence. Sixty of 541 patients with pathologically confirmed NPC were included in the present study from the Department of Radiation and Medical Oncology, Zhongnan Hospital of Wuhan University the from November 2011 to July 2018. Inside-field recurrence was defined as 95% or more of the r-GTVnx or r-GTVnd volume within the 95% isodose of PTVnx or GTVnd 20. The detailed sites of local recurrence were judged by three senior attending radiation oncologists.

### Cell cultures and sorting of cell populations

The CNE-2 cell line was presented from Prof. Yunfei Xia at Sun Yat-sen University Cancer Center (Guangzhou, China)^21^. CNE-2 cell was cultured in RPMI-1640 medium (Hyclone, Logan, UT, USA) with 10% fetal bovine serum (Hyclone), 100 units/ml penicillin and 100 mg/ml streptomycin (Hyclone). The Sorting of cell populations was referred to a previously reported method 22. Sorted CNE-2s cell was cultured in DMEM/F12 (1:1) medium (Hyclone) with 20μg/L EGF (PeproTech, Rocky Hill, USA), 20μg/L bFGF (PeproTech) ,2% B27(no vitamin A, Gibco, Carlsbad, Calif, USA),100 units/ml penicillin and 100 mg/ml streptomycin (Hyclone). All the two cell lines were cultured in a 37℃ incubator (Sanyo, Japan) with 5% CO_2_. Cells were digested by 0.25% trypsin and 0.02% EDTA solution (Sigma, St. Louis, MO, USA).

### Colony formation assay

A colony formation assay was used to analyze cell renewal ability and evaluate radio- sensitivity. Viable CNE-2s and CNE-2ns cells (100, 200, 400, 800, 1000 and 2000 cells/well) were seeded in 6-well plates. Once cell adhered, 1μM CQ (Sigma, St. Louis, MO, USA) and 10μM zinc (Sigma) were added to the medium. After incubation for 6 h, the plates were irradiated with 0, 1, 2, 4, 6, 8 and 10 Gray (Gy) sequentially with the Small Animal Radiation Research Platform (SARRP, PXI X-RAD 225Cx, CT, USA) from a 204-kV photon beam. After 10 days, the colonies were fixed with 4% paraformaldehyde (PFA) and stained with crystal violet. The cells were photographed and the numbers of colonies were counted. A “multitarget-single hitting” model was applied to fit the survival curve.

### Flow cytometry

The CNE-2s cells were placed in 6-well plates were starved for 6 h and treated with 1μM CQ and 10μM zinc for 24 h. The cells were harvested, washed twice with cold PBS, and stained with FITC-conjugated annexin V for 20 min and propidium iodide for 5 min (Sigma-Aldrich) in the dark. The stained cells were assessed by flow cytometry (FACSAria ^TM^[Ⅲ], BD, Mountain View, USA)), and analyzed by FlowJo vX.0.7 software. The intracellular apoptosis rates were measured by flow cytometry as previously described 20. Briefly, 5
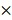
10^5^ cells were seeded in 6-well plates and then treated as indicated. The cells were washed twice with cold PBS and collected for fluorescence analysis using a flow cytometer (FACSAria TM[Ⅲ], BD).

### Western blotting analysis

Western blotting was performed as previously described 23. All the antibodies for western blotting were purchased from Cell Signal Technology (Danvers, MA, USA). The immunoreactive proteins were detected by enhanced chemiluminescence and quantified with image Pro Plus 6.0.

### Immunofluorescence staining

The CNE-2s cells were seeded on 10 mm coverslips, fixed with 4% PFA for 30 min, treated by 0.1% Triton X-100 and blocked in 5% BSA for 1 h at room temperature. Sequentially the fixed cells were incubated with primary antibody at 4 °C overnight, then washed with PBST and incubated with FITC-labelled secondary antibody for 1 h at room temperature. The nuclei were labelled with DAPI (2 mg/ml), and the immunofluorescence (IF) staining was analyzed using a fluorescence microscope (Olympus IX 73 DP80, Japan). The mean destiny was applied to semi-qualified by Image Pro Plus 6.0.

### Autophagosome and autophagy detection

Transmission electron microscopy (TEM) performed with an H-600IV microscope (Hitachi, Tokyo, Japan) was utilized to analyse ultra-structural images of autophagic vacuoles, auto-phagosomes and autolysosomes by method previously reported^24^. Acridine orange staining method was referred to our previous studies 18. A GFP-LC3 lentivirus (Genechem, Shanghai, China) was used to facilitate the detection of autophagy. This procedure was performed in accordance with the manufacturer's instructions (Genechem).

### Immunohistochemical staining

Samples from patients and xenograft tumors were fixed using formalin and then used for immunohistochemical staining to measure different protein expression. Tissues were dehydrated in graded ethanol solutions, cleared in xylene, embedded in paraffin and placed onto slides. The slides were incubated with the appropriate antibodies. The Immunohistochemical (IHC) staining was analyzed using a microscope (Olympus IX 73 DP80, Japan). The mean destiny was applied to semi-qualified by Image Pro Plus 6.0.

### Animals and* In vivo* assay

Six-week-old female BALB/C nu/nu mice were purchased from Beijing Vital River Laboratory Animal Technology (Beijing, China) and all animal experiments were performed according to the Wuhan University Animal Care Facility and National Institutes of Health guidelines. For the tumorigenesis ability test, freshly sorted CNE-2s cells suspended in 200 mL PBS were inoculated into the flanks of 6- to 7-week-old BALB/c-nu/nu mice. The mice were monitored 10 days for palpable tumor formation and euthanized 14 days after transplantation to assess tumor formation. Tumors were measured using a Vernier caliper, weighed, and photographed.

For the synergic effect test of CQ combined with Zinc and IR, the method was described as follows. Approximately 1
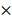
10^5^ CNE-2s cells were harvested, resuspended in 100 ml PBS, and injected subcutaneously into the right flank of each mice. Treatment was commenced when the tumor size reached approximately 200 mm3. The mice were randomized into 6 groups and treated as follows: CQ combined with zinc (10 mg/kg CQ + 20 mg/kg zinc i.p. 30min before radiotherapy), radiotherapy (30Gy/3f, irradiation once every other day for one-week, Small Animal Radiation Research Platform SARRP, PXI X-RAD 225Cx, CT, USA), CQ + zinc combined with radiation , rapamycin + CQ + zinc combined with radiation (3 mg/kg rapamycin + 10 mg/kg CQ + 20 mg/kg zinc i.p. 30min before radiotherapy) and saline solution as an untreated vehicle. The size of the subcutaneous tumors and weight of the mice were recorded every 2 days. Before the mice were radiated, we applied X-rays fluoroscopy to confirm the completed foci in the irradiated field. Tumor volume (V) was calculated according to the formula: Π/6 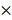 length 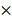 width^2^.

### Statistical analysis

Unless stated otherwise, all experiments were conducted in triplicate. Data was expressed as the mean ± SD of at least 3 independent experiments. The significance of differences between mean values was determined using two-way ANOVA. P value less than 0.05 was considered significant.

## Results

### Clinical characteristics of NPC patients

Sixty patients were included in this study finally. Twenty NPC patients experienced inside-field recurrence after initial definitive radiotherapy treatment, all of them received re-biopsy and were confirmed with pathological diagnosis. Forty matched patients were relapse free with follow up duration more than 3 years. The median follow-up time was 44.9 months (range 30.2 to 64.5 months), and the median age was 47 years (range 31 to 64 years). All patients exhibited non-keratinizing squamous cell carcinoma. The baseline characteristics were shown in Table 1.

### High expression levels of CD44, autophagy-related proteins (Beclin1 and LC3) in NPC patients with inside-field recurrence

**Fig 1:**
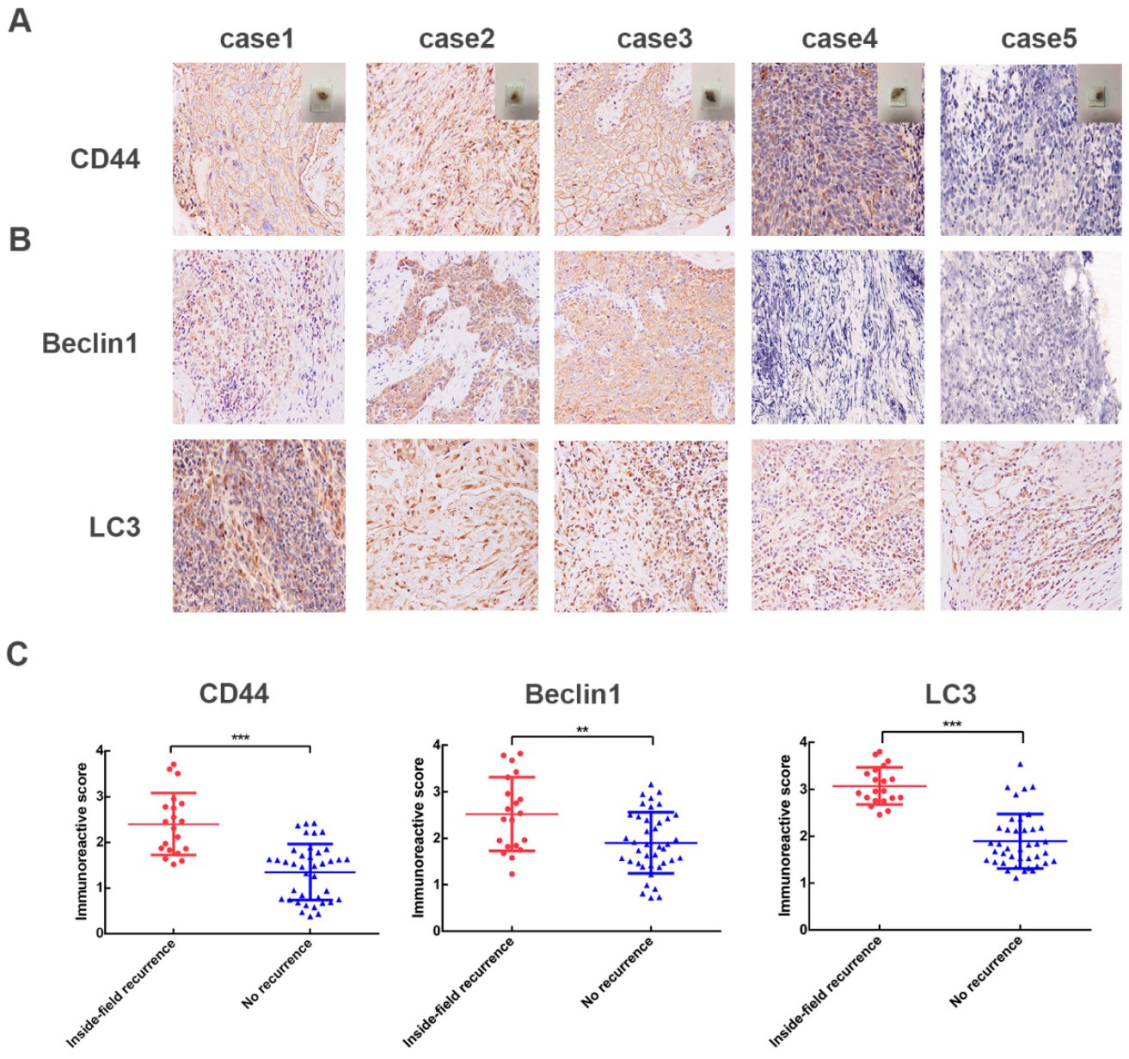
High levels of CD44, Beclin1, LC3 in 60 NPC patients. (A) Representative IHC images of CD44; case 1-3 represent NPC patients with inside-field recurrence and case 4-5 represent ones with relapse free. (B) Representative IHC images of Beclin1 and LC3. (C) Scatter plot of the protein levels of CD44, Beclin1, LC3 for the NPC patients with and without inside-field recurrence. Immunoreactivity was analyzed in 5 random areas for each pathological section and was scored as 0+(no staining), 1+(weak staining), 2+(moderate staining),3+(strong staining),4+ (very strong staining). (*, P<0.05; **, P< 0.01; ***, P<0.001).

We then adopted immunohistochemical assays to examine the protein expression pattern of CD44, Beclin1 and LC3 in 60 NPC cases. Compared with the relapse free NPC patients, NPC patients with inside- field recurrence had higher levels of CD44 proteins in tumor tissues (Fig. 1A). Furthermore, the expression levels of Beclin1, LC3 proteins, which presented high autophagy activity, were significantly higher in NPC patients with inside-field recurrence (Fig. 1B). Fig. 1C showed scatter diagrams of the protein levels of CD44, Beclin1, LC3 for the NPC patients with and without inside-field recurrence. The results implicated the existence of cancer stem cells and increased autophagy activity played critical roles in the inside-field recurrence of NPC.

### Successfully establishing and characterizing of CNE-2 stem-like cells

**Fig 2:**
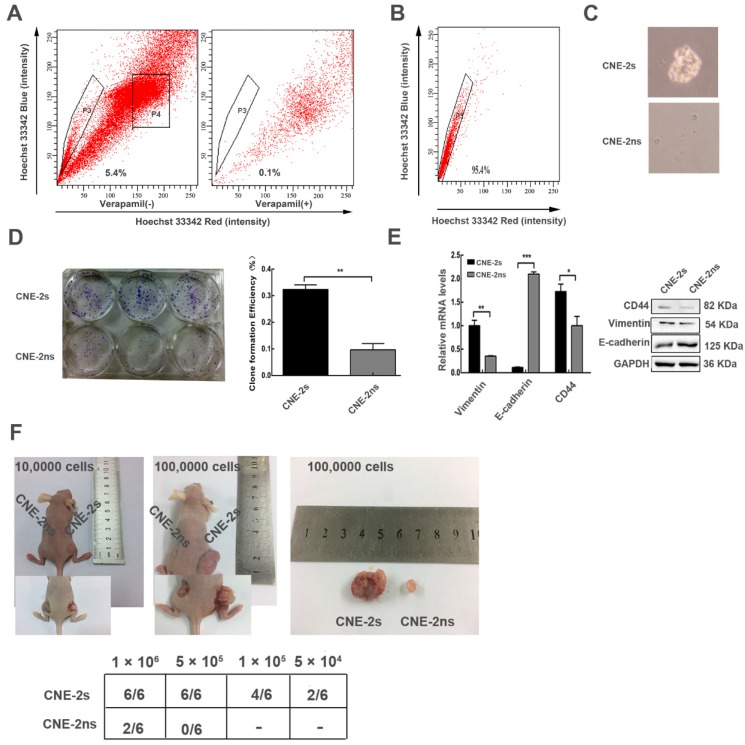
Side population cells were isolated from nasopharyngeal cancer cell (CNE-2) and characterized with stem cell properties. (A) sorting of CNE-2 cells by flow cytometry. (B) the sorting purity of the freshly sorted SP cells. (C) the CNE-2s and CNE-2ns cells were seeded in tumor sphere culture medium. (D) clone formation results and statistical analysis. (E) the CNE-2s and CNE-2ns cells lysates were analyzed by Western blotting and Realtime-qPCR with anti-CD44, anti-E-cadherin and anti-Vimentin. GAPDH served as a loading control. (All data represent the mean ± SD; n=3; *, P<0.05; **, P< 0.01; ***, P<0.001) (F) the indicated amounts of CNE-2s and CNE-2ns cells were transplanted into 6 weeks BALB/c-nu mice.

Next, we attempted to isolate cancer stem-like cells from CNE-2 cell line by flow cytometry. The sorting referred to previous reports 22. CNE-2s cells occupied 5.4% of the total cells (Fig. 2A). CNE-2s (P3) and CNE-2ns (P4) cells were collected for subsequent experiments; the purity of CNE-2s cells was 96% (Fig. 2B). The sorted CNE-2s and CNE-2ns cells were cultured in serum-free medium, CNE-2s cells showed clear microsphere formation after 5 days, while CNE-2ns cells were still scattered and grew slowly (Fig. 2C). The Clone Formation Efficiency (CFE) of CNE-2s and CNE-2ns cells was (32.33 ± 3.055) % and (9.67 ± 4.041) % respectively. There was a significant difference in CFE between the two groups (P<0.001; Fig. 2D). The mRNA and protein expression levels of stem cell-related gene CD44, interstitial cell-related genes E-cadherin and Vimentin were also detected in two cell lines. Results were shown in Fig. 2E. Compared with CNE-2ns cells, the mRNA and protein expression of CD44 and Vimentin in CNE-2s cells were higher (P<0.05 for CD44 and P<0.01 for Vimentin), E-cadherin were lower (P<0.001). To further explore whether CNE-2s cells had stem-like cells activity, the same concentrations of CNE-2s and CNE-2ns cells were inoculated subcutaneously in the left and right hind legs of BALB/c-nu female nude mice. The tumors volume of CNE-2s cell was also significantly larger than that of CNE-2ns cells with the same concentration of tumor cells, suggesting that the sorted CNE-2s cells had stem-like cells activity and could be used as models for stem cells research. The expression of CD44, Vimentin and E-cadherin were consistent with our *in vitro* results and there was no significant difference in the expression of Beclin1 and LC3 in these two tissues (Additional file 2: Fig. S1).

### CNE-2s cells are resistant to radiotherapy through activation of autophagy

**Fig 3:**
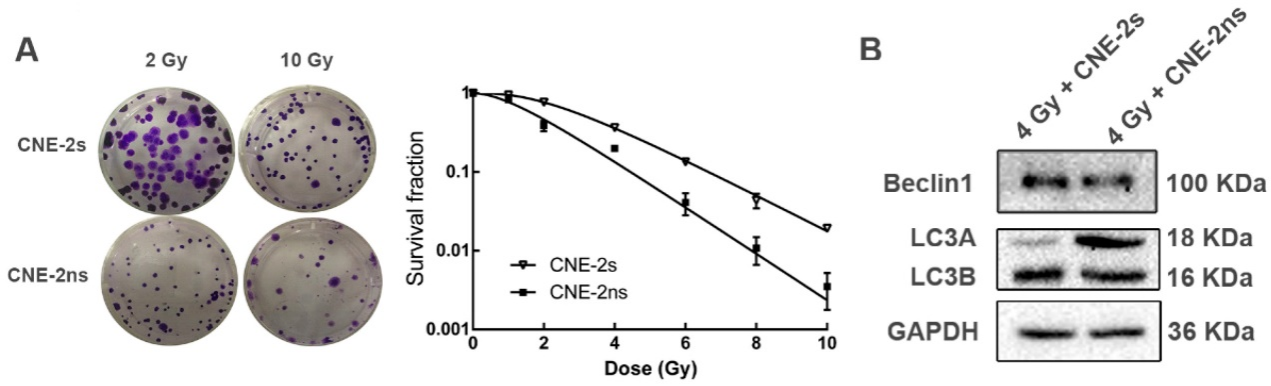
Comparison of radiosensitivity and autophagy levels in CNE-2s and CNE-2ns cells. (A) colony-formation assay for CNE-2s and CNE-2ns cells using irradiated (2 Gy and 10 Gy). (B) dose-survival curves of irradiated CNE-2s and CNE-2ns cells (range, 0-10 Gy. Data represent the mean ±SD; n=3). (C) Western blotting detection of Beclin1, LC3A and LC3B in CNE-2s and CNE-2ns cells assessed 24 hours after 4 Gy IR.

We then explored the association between autophagy and radioresistance *in vitro*. After different doses of ionizing radiation (IR) treatment, the colony formation rates of both were counted. The survival fraction of CNE-2s cells was higher than that of CNE-2ns cells at the same dose (Fig. 3A), and the SF2 value was significantly higher than that of CNE-2ns cells (0.617± 0.012 and 0.180±0.011, P<0.01). After 4 Gy IR treatment, total cellular protein was extracted and the autophagy levels were observed. It was found that the expression levels of autophagy-related proteins Beclin1 and LC3 in CNE-2s cells were significantly higher than that in CNE-2ns cells (Fig. 3B). The results showed that CNE-2s cells might be realized radioresistance through activation of autophagy.

### CQ combined with zinc enhances radiosensitivity of CNE-2s cells through the inhibition of autophagy

**Fig 4:**
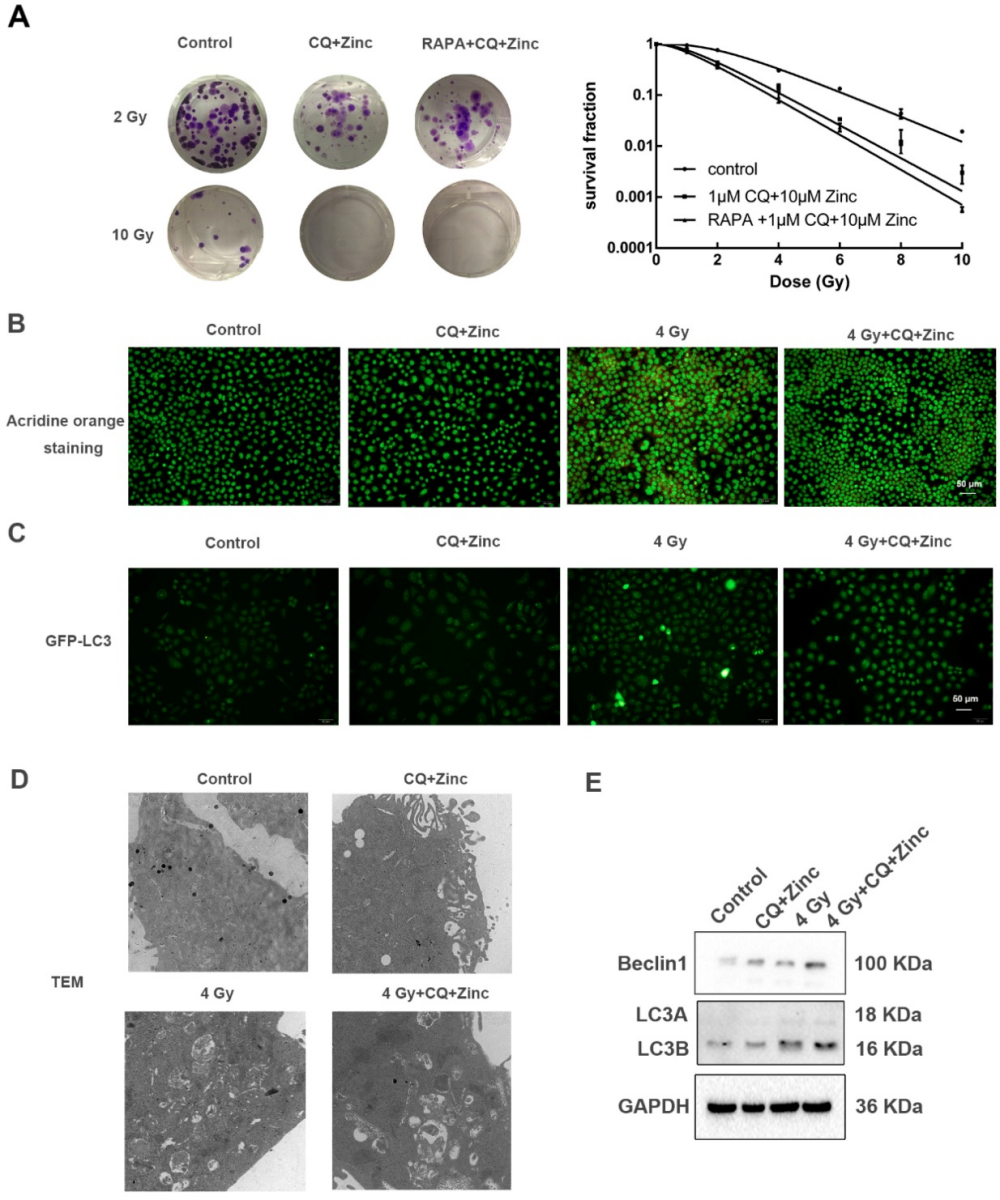
CQ combined with zinc enhanced the radiosensitivity of CNE-2s cells. (A) Representative crystal violet staining photos of CNE-2s cells irradiated with 2 Gy and 10 Gy. All values shown were mean ± SD of triplicate measurements and repeated three times with similar results. Clone formation assay was used to detect the radiosensitivity of CNE-2s cells with different treatments. (B) Representative images of acridine orange staining in CNE-2s. (C) Representative images of GFP-LC3 tracer protein. (D) Representative images of transmission electron microscopy to observe the structure and number of autophagosomes. (E) Western blotting analysis of Beclin1, LC3A and LC3B protein levels in CNE-2s cells.

In order to determine whether CQ combined with zinc could increase sensitivity of CNE-2s cells to radiation, the colony formation assay was used. The results were shown in the Fig. 4A. Survival fraction of 1 μM CQ combined with 10 μM zinc administration was lower than IR alone (IR group). There was no significant difference in survival fraction between the groups of CQ combined with zinc and one with pretreated with rapamycin for two hours. Acridine orange staining and GFP-LC3 were introduced to detect the autophagy. Four Gy IR could enhance the intensity of orange-red fluorescence in CNE-2s cells. However, CQ combined with zinc could decrease the intensity of orange-red fluorescence induced by 4 Gy IR in CNE-2s cells (Fig. 4B). The GFP-LC3 fusion protein particles were transferred into CNE-2s cells and then given different treatments. After 24 hours the number of fluorescent spots in the 4 Gy group was increased compared with the control group (Fig. 4C). Subsequently, we observed the structure and the number of autophagosomes in different treatment groups using transmission electron microscope. It was found that autophagosomes were mainly monolayer and bilayer membrane structures, containing some cytoplasmic components. Four Gy IR could increase the numbers of autophagosomes. In the group of CQ combined with zinc with 4 Gy, the autophagosomes became more monolayer and larger in size (Fig. 4D). The expression of Beclin1 and LC3II/LC3I proteins in the 4 Gy treated group was higher than that in the control group (Fig. 4E). These results indicated that CQ combined with zinc could inhibit autophagy to realize radiosensitization of CNE-2s cells.

### CQ combined with zinc inhibits DNA-damage repair response through NF-κB signal pathway

**Fig 5:**
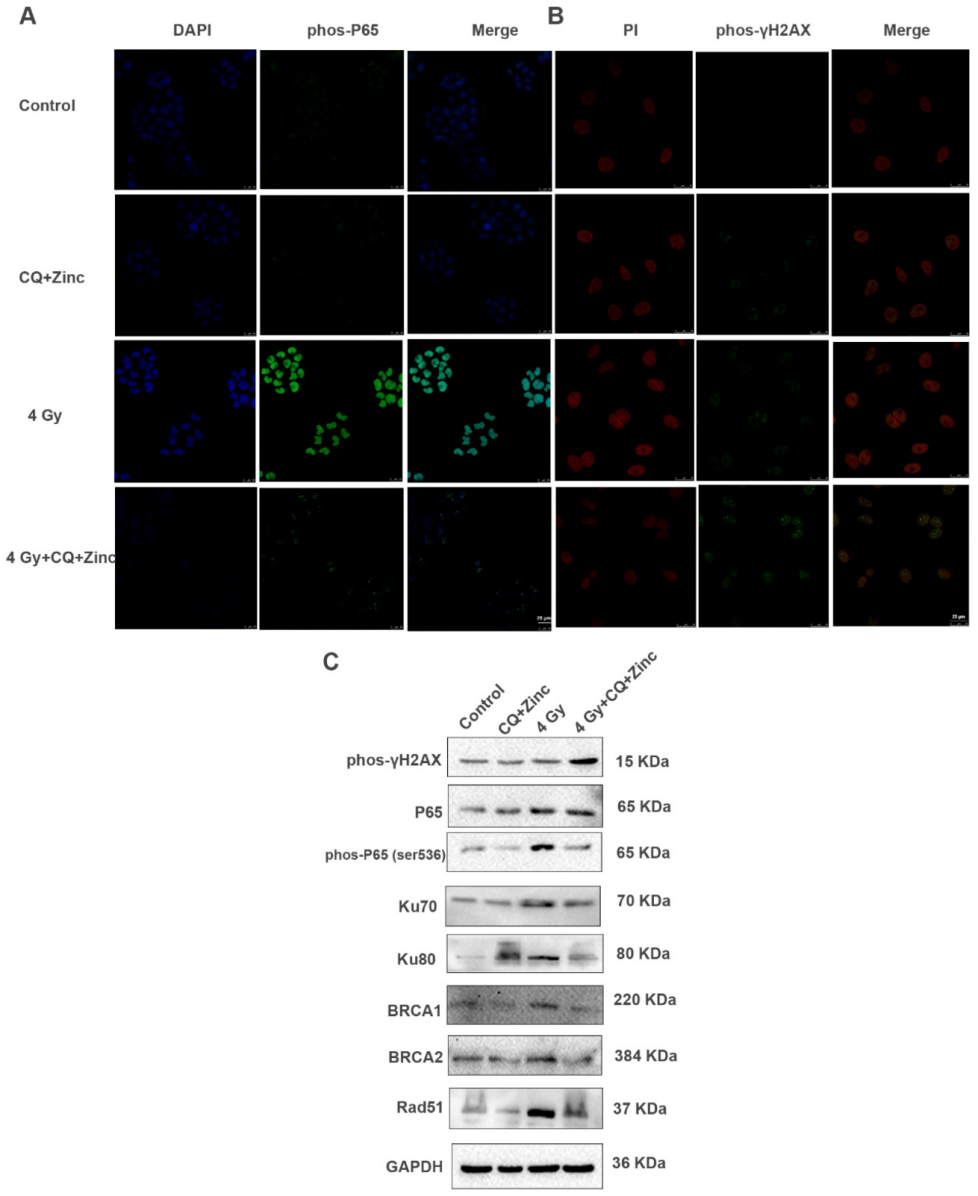
CQ combined with Zinc suppressed NF-κB activity and increased radiation induced DNA damage. (A) Immunofluorescence staining localization of phospho-p65(phos-P65) in different treatment groups of CNE-2s cells. (B) Immunofluorescence staining of phos-γH2AX. (C)Western blotting analysis of key proteins involved in DNA damage and repair in different treatment groups.

IR activates NF-κB activity and initiates DNA repair processes. We have reported that CQ combined with zinc could downregulate the NF-κB activity in many human cancer cell lines 19, 23. Four Gy IR could significantly enhance the fluorescence intensity of phosphorylated P65 in the nucleus of CNE-2s cells compared with the control group, and CQ combined with zinc administration could reverse the translocation of phosphorylated P65 induced by 4 Gy in CNE-2s cells (Fig. 5A).Beyond that, irradiation could also induce DNA damage, mainly DNA double-strand breaks (DSB) and γH2AX is considered a specific DSB marker. We assessed phos-γH2AX foci by IF as shown in Fig. 5B. The CQ combined with zinc group showed a higher mean fluorescence intensity than the control group after 24 hours of radiation exposure. At the same time, we also detected the expression levels of DNA damage and repair proteins and found that the expression of phosphorylated P65, Ku70, Ku80, BRCA1, BRCA2, and Rad51 in the group of CQ combined with zinc with 4 Gy was significantly decreased compared with the 4 Gy group, and the phos- rH2AX expression level in the group of CQ combined with zinc with 4 Gy was much higher than that in the 4 Gy group (Fig. 5C). Results indicated that CQ combined with zinc prolonged DNA damage after radiation, which led to failure in DNA damage repair.

### CQ combined with zinc induces apoptosis of CNE-2s cells to IR

**Fig 6:**
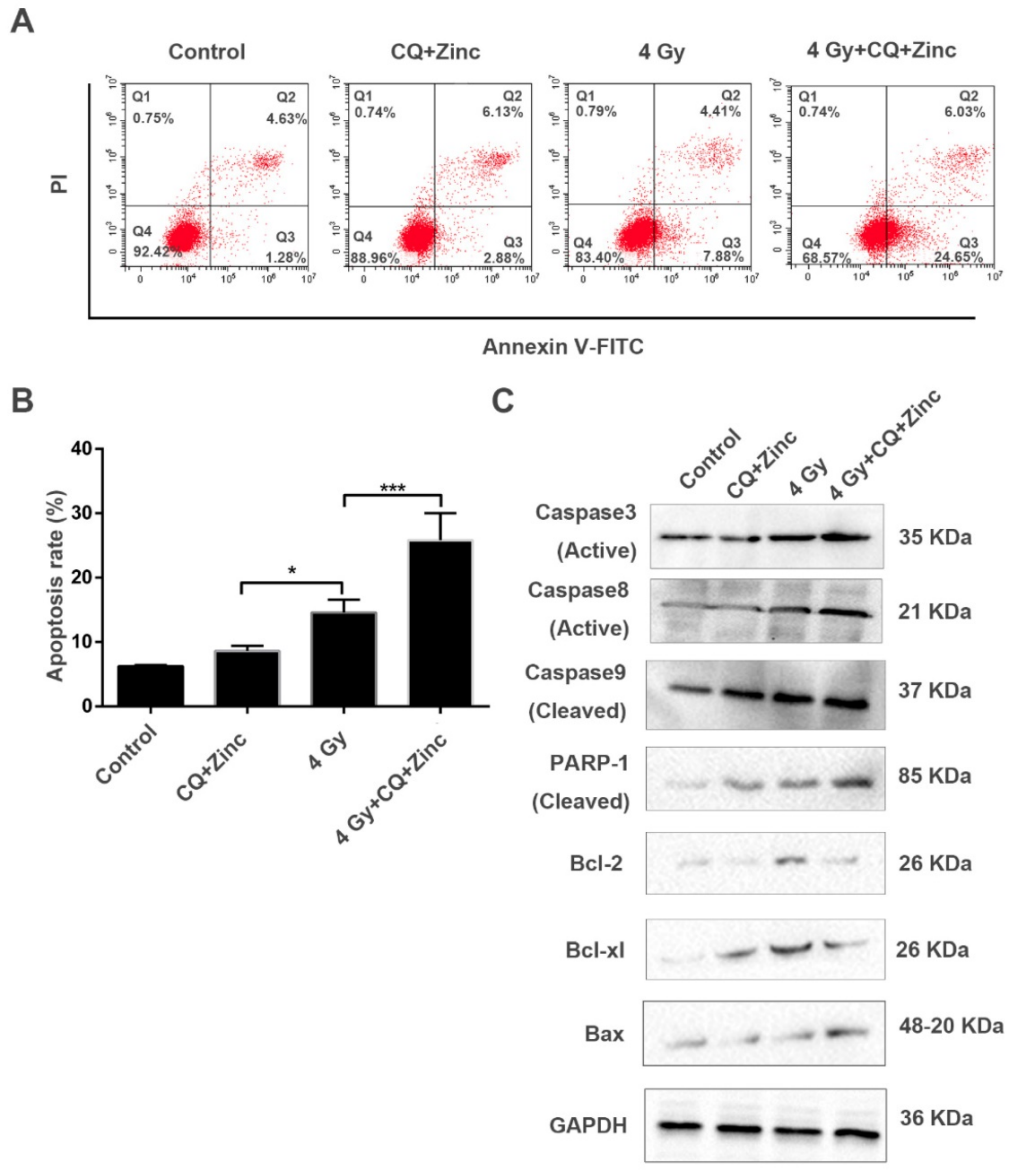
CQ combined with zinc promoted radiation-induced apoptosis in CNE-2s cells. (A) cell cytometry analysis of Annexin V and PI for apoptosis in CNE-2s cells. (B)the administration treatment group had significantly more cell apoptosis than individual ones. Data were expressed as mean ± SD, n=3, * p<0.05, ***p<0.001. (C) Western blotting analysis of apoptosis-related proteins in different treatment groups.

To further analyze the mechanisms of the synergic effect of CQ+Zinc with IR in CNE-2s cells, apoptosis of CNE-2s cells was detected by flow cytometry in different treatment groups. Our results showed that the apoptosis of 4Gy group was increased compared with CQ combined with zinc administration alone (P<0.05). The administration combined with 4 Gy IR was significantly higher percentage of apoptosis than other groups (P<0.001, Fig. 6A and 6B). IR induced apoptosis through the activation of caspase-3 in CNE-2s cells, CQ combined with zinc pretreatment could further activate caspase-3, caspase-8, caspase-9, PARP cleavage and Bax, suggesting that CQ combined with zinc enhanced radiation-induced apoptosis of CNE-2s cells through mitochondria and caspase-dependent pathway (Fig. 6C).

### CQ combined with zinc increased radiosensitivity of CNE-2s cell *in vivo*

**Fig 7:**
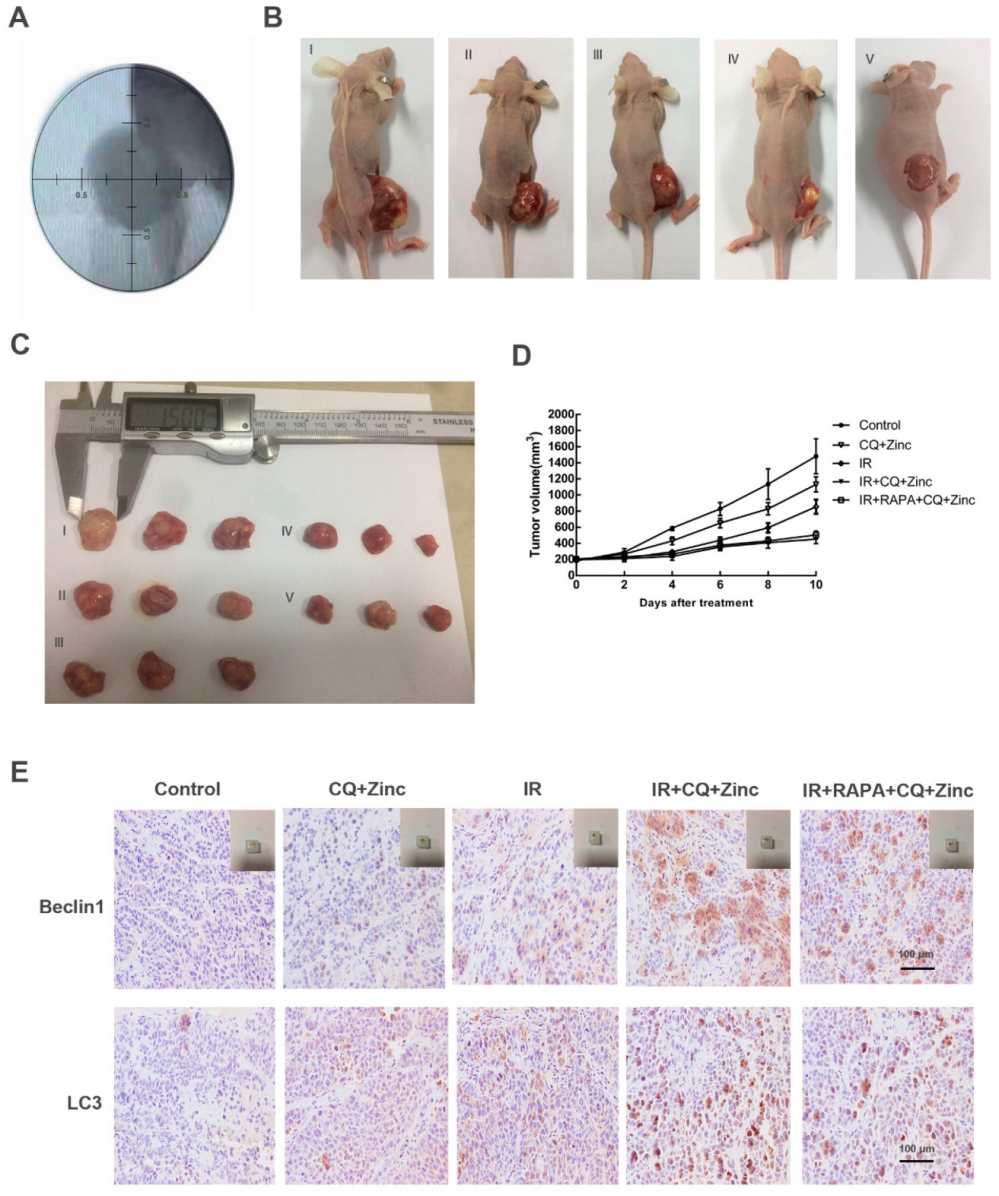
CQ combined with zinc inhibited tumor growth* in vivo*. (A) Simulation position before IR. (B) Representative image of tumor growth in different treatments (I, II, III, IV, V represent control, CQ+Zinc, IR, IR + CQ + Zinc, IR + RAPA + CQ +Zinc respectively). (C) Gross view of tumor. (D) Growth curve of tumor volume. (E) Representative immunohistochemistry staining images of Beclin1 and LC3 in tumor tissue.

To investigate the antitumor effects of CQ combined with zinc* in vivo*, we examined the therapeutic potential of CQ combined with zinc alone or combined with IR. The results were shown in Fig. 7. The tumor size was slightly decreased in the simple drug-administered group compared with the control group (p<0.05). The tumor volume of the drug-combined X-ray group was significantly smaller than that of the IR alone group, and there was no significant difference in the rapamycin combination group (Fig. 7B, 7C and 7D). The results of immunohistochemistry of tumor tissue showed that the expression of autophagy protein in the drug-combined with IR group was higher than that of the radiotherapy alone group, and there was no significant difference between the rapamycin group and the radiotherapy group (Fig. 7E).

## Discussion

Radiotherapy is the most commonly applied treatment for nasopharyngeal carcinoma (NPC) 25, but we found clinically that some patients experienced tumor recurrence in a short time, which is universal due to radiotherapy resistance. To find out the reason, we collected twenty nasopharyngeal carcinoma patients diagnosed with local inside-field recurrence from our department before July 2018. Through detecting the expression of stem cells and autophagy related proteins in tumor tissues of these patients, we found some interesting results. Some patients who had inside-field recurrence had higher expression of CD44, autophagy-related proteins. There were reports about the existence of cancer stem cells (CSCs) in NPC 7, 22 and CD44 was considered as one of potential surface markers of NPC stem cells 26, 27. Hence, we believed that inside-field recurrence of NPC might be related to presence of CSCs and autophagy activity. Based on this, we proposed a hypothesis whether it could increase the sensitivity of NPC to radiotherapy by inhibiting autophagy of CSCs.

In order to confirm our assumption, We sorted CNE-2 stem-like (CNE-2s) cells and CNE-2 non-stem-like (CNE-2ns) cells from CNE-2 cells which were the most poorly differentiated NPC cell line^26^, ^28^. CSCs possessed the ability to initiate tumor growth and sustain self-renewal as well as metastatic potential 22, 29. These characteristics were used to identify the presence of stem-like cells.* In vitro* experiments, our results showed that CNE-2s cells had stronger self-renewal activity than CNE-2ns cells. It is known that epithelial-mesenchymal transition (EMT) is closely related to increase the stemness and metastasis of cancer cells 30. In our study, expressions of EMT-related genes and proteins in CNE-2s cells were higher than CNE-2ns cells, suggesting that CNE-2s cells had higher metastasis activity. Above results proved that the sorted CNE-2s cells had properties of CSCs and the CSCs model was successfully established. Subsequently, we compared the radiosensitivity and autophagic activity of the two cells. We further discovered and identified the radioresistance and enhanced autophagy activity of NPC CSCs induced by radiation.

As has been previously reported, the maintenance of stemness characteristics is closely related to autophagy activity 31. And autophagy may play a critical role in determining radiosensitivity of cancer cells 31, 32. To determine whether the increased radiation sensitivity in CNE-2s resulted from autophagy inhibition, we introduced autophagy activator rapamycin, which could upregulate autophagy activity of cancer stem cells after IR 33, we applied rapamycin in addition to the administration of CQ combined with zinc and IR on CNE-2s cells. As shown in the Results section, the triple treatment group (IR + Rapamycin + CQ +Zinc) had an increased radiosensitivity compared with IR alone, but there was no statistically significant difference compared with the double treatment group (IR + CQ + Zinc). Based on these results, we speculated that CQ combined zinc achieved radiosensitivity through the inhibition of autophagy. To confirm the observed link between CQ + Zinc and autophagy, we detected autophagy activity of CNE-2s cells using acridine orange (AO) staining, transmission electron microscope (TEM), GFP-LC3 tracer and western blotting. Our results showed that IR could significantly induce autophagic change and increase LC3-II protein levels in NPC CSCs. When NPC cells were pretreated with CQ combined with zinc *in vitro*, autophagy activity was markedly decreased. Hence, we showed that CQ combined with zinc might inhibit autophagy activity induced by IR, which resulted in an increased radiosensitivity in CNE-2s.

Our previous study demonstrated that CQ combined with zinc could inhibit NF-κB activation induced by IR in HeLa cells 23.It is well known that NF-κB plays a critical role in cellular protection against a variety of apoptotic stimuli, including DNA damage, and inhibition of NF-κB leads to radiosensitization 34. Blocking radiation-induced NF-κB activation has been shown to increases apoptotic response and decreases growth and clonogenic survival of several human cancer cells 35-37. Upon activation, NF-κB translocates into the nucleus, binds sequence-specifically to the promoter/enhancer region of various DNA repair genes and transactivates their expression 38. This might allow the cancer cells that are surviving the radiation exposure to develop a clone (clonal selection), re-grow (tumor cell proliferation and growth), and cause tumor relapse 39. Our current study indicated that IR would induce activation of NF-κB and increase NF-κB DNA-binding activity. However, CQ combined with zinc could prevent NF- κB/P65 translocation to the nucleus and decrease the expression of several proteins encoded by NF-κB dependent genes. IR could induce various forms of DNA damage include breaking the bases and cleavage of the DNA backbone, resulting in DNA single strand breaks (SSBs) and double strand breaks (DSBs) 40. γH2AX is a highly specific and sensitive molecular marker for monitoring both DSBs initiation and resolution^41^.We found that phosphorylation of histone γH2AX was enhanced by combination treatment with CQ combined with zinc and 4 Gy compared with IR alone, suggesting that CQ combined with zinc might be blocking DNA damage-repair pathways induced by IR in CNE-2s cells. Two main pathway are responsible for DNA DSB repair, which are NHEJ and HR 42.In this study, we found that CQ combined with zinc remarkably down-regulated the key proteins including BRCA1, BRCA2, RAD-51 (HR pathway) as well as Ku70 and 80 (NHEJ pathway) in NPC stem-like cells, suggesting radiosensitization induced by CQ combined with zinc might be mediated by suppression of DNA damage repair 43.

Radiotherapy prolongs patient's survival through decreasing proliferative capacity and killing tumor cells 44. However, repopulation of tumor cells during or after IR is an important obstacle to achieve the desired response 45. Prominent hallmark of cancer are apoptosis evasion and the ability to self-govern and proliferation 46. Following radiation, cancer cell death may occur mainly through necrosis, autophagy and apoptosis 47. Autophagy is induced in response to many stresses that ultimately lead to apoptosis 48. NF-κB induces activation of apoptosis inhibitor. IR might promote apoptosis pathway through activation of the caspase cascade. Recruit of caspase inhibitor with radiation to stimulate autophagy pathway may be a useful strategy to increase survival times of patients and a remarkable delay in tumor recurrence 49. Our results showed that CQ combined with zinc increased IR-induced apoptosis, indicating its potential roles of inhibitor.

In this study, we demonstrated that the presence of cancer stem cells and activated autophagy might be the cause of inside-field recurrence of NPC patients after definitive radiotherapy. CQ combined with zinc had the effect of radiosensitization for NPC CSCs* in vitro* and *in vivo*. Mechanistically, clioquinol combined with zinc could inhibit inherent and IR-induced autophagy activity, delay DNA damage repair and induce apoptosis in NPC stem-like cells. This study provided an important and new therapeutic direction for recurrence NPC patients.

## Supplementary Material

Supplementary figure.

## Figures and Tables

**Table 1:** Clinical characteristics of NPC patients (n = 60)

Characteristics	Inside-field recurrence (n = 20)	Without recurrence (n = 40)
	No. of cases (%)	No. of cases (%)
**Gender**		
Male	15(75%)	30(75%)
Female	5(25%)	10(25%)
**WHO Histologic type**		
IIa	4(20%)	8(20%)
IIb	16(80%)	32(80%)
**T classification**		
T1	2(10%)	5(13%)
T2	8(40%)	16(40%)
T3	7(35%)	15(38%)
T4	3(15%)	4(9%)
**N classification**		
N1	1(5%)	6(15%)
N2	16(80%)	28(70%)
N3	3(15%)	6(15%)
**Clinical stage**		
I	0(0%)	0(0%)
II	1(5%)	2(5%)
III	14(70%)	28(70%)
IVa+IVb	5(25%)	10(25%)
**Neoadjuvant chemotherapy**		
Yes	17(85%)	32(80%)
No	3(15%)	8(20%)
**Concurrent chemotherapy**		
Yes	13(65%)	25(62%)
No	7(35%)	15(38%)
**PTV-GTVt dose**		
≥70Gy	18(90%)	36(90%)
<70Gy	2(10%)	4(10%)
**Radiotherapy modality**		
IMRT	20(100%)	40(100%)
3D-CRT or 2D	0	0

## Abbreviations

NPC: Nasopharyngeal carcinoma
FACS: Fluorescence-activated cell sorting
CSCs: Cancer stem cells
Bcl-2: B cell Lymphoma-2
SP: Side population cells
Bax: Bcl-2 Associated X protein
NSP: Non-side population cells
PARP-1: Poly (ADP-ribose) Polymerase
γ-H2AX: γ-H2A Histone Family Member X
CQ: clioquinol
AO: Acridine orange
BRCA1: Breast Cancer Susceptibility Genes 1
TEM: Transmission Electron Microscope
RAD51: RAD51 Recombinase
IR: Ionizing radiation
Caspase: Cysteine-aspartic Proteases
IF: Immunofluorescence
DSBs: DNA double-stand breaks
IHC: Immunohistochemistry
