# Supplementary figures and images for: Radiosensitization of Clioquinol Combined with Zinc in the Nasopharyngeal Cancer Stem-like Cells by Inhibiting Autophagy in Vitro and in Vivo
Source: Int J Biol Sci. 2020 Jan 14;16(5):777–89. doi: 10.7150/ijbs.40305 (PMC7019136; doi:10.7150/ijbs.40305)

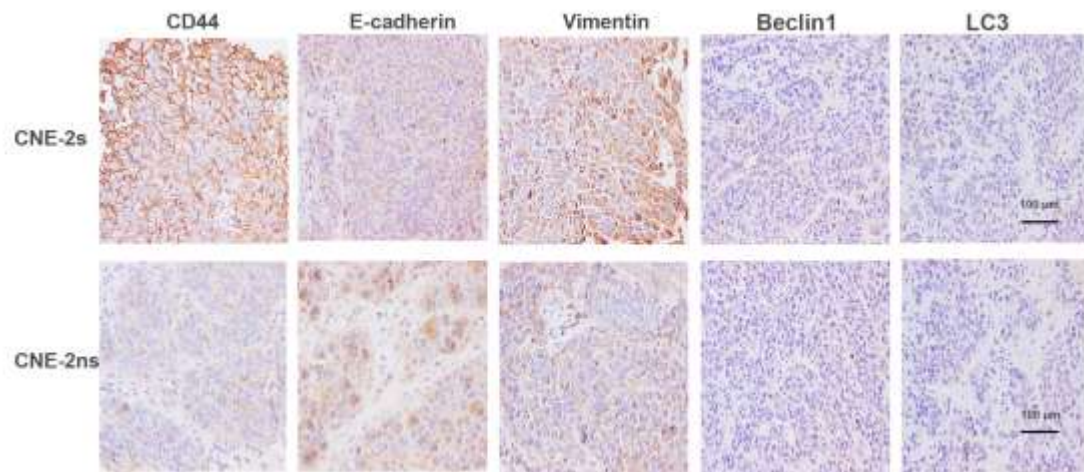

**Fig.S1:** The expression of CD44, Vimentin, E-cadherin, Beclin1 and LC3 in CNE-2s and CNE-2ns tumor tissues.

Supplement: Supplementary file 1 — Supplementary figure. [file ijbsv16p0777s1.pdf]
